# Dietary diversity (DD) and associated factors among Lactating women (LW) in Pawie district, Northwest, Ethiopia, 2019: community-based cross-sectional study

**DOI:** 10.1016/j.heliyon.2021.e08495

**Published:** 2021-11-27

**Authors:** Sileshi Mulatu, Habtamu Dinku, Chalachew Yenew

**Affiliations:** a Department of Pediatrics and Child Health Nursing, College of Medicine and Health Sciences, Bahir Dar University, Bahir Dar, Ethiopia; b Department of Public Health, College of Health Sciences, Debre Tabor University, Debre Tabor, Ethiopia

**Keywords:** Lactating women, Dietary diversity, Household Food Insecurity Access Scale, Pawie, Ethiopia

## Abstract

**Background:**

Low Dietary Diversity (DD) result in severe problem among the vulnerable group in low-income countries (LICs), whose diets are predominantly starchy staples. Lactating Women (LW) from LICs are considered a nutritionally vulnerable group. It results in many consequences on the health and well-being of children, households, communities, and the nation. However, there is little empirical evidence on factors contributing to low DD among LW in Ethiopia and the proposed study site. Therefore, this study aimed at assessing the DD and associated factors among LW in Pawie district, Northwest Ethiopia.

**Methods:**

A Community-based cross-sectional study was conducted among 806 LW from March to May 2019 G.C. DD assessed using 24 h dietary recall methods with structured questionnaires.

A mean dietary diversity score (DDS) was computed for ten food groups. Food insecurity measured using a 9-item Household Food Insecurity Access Scale (HFIAS). The multivariable logistic regression model was used to see the relevant associations. The variables which have a significant association with DD were identified based on AOR, P-value ≤ 0.05, and 95% Cl.

**Results:**

A total of 806 LW aged 15–49 years were interviewed with a response rate of 100%. About two-third of LW had low DD (<5 food groups). Fathers occupation being daily laborer [AOR = 1.82, 95% CI (.339, 9.784)], birth interval less than 24 months [AOR = 3.7, 95 % CI (1.743, 7.885)], family size greater than six members [AOR = 1.55, 95 % CI (1.046, 2.313)] and food insecurity [AOR = 2.23, 95 % CI (1.626, 3.066)] were more likely associated with the low DD among LW compared to their counterpart.

**Conclusion:**

The DD among LW was low. Low Dietary Diversity was statistically associated with low birth intervals, large family sizes, and food insecurity. Hence, attention should be paid to the identified factors of low DD of LW to improve their health, and that of their children as well as their family.

## 1 Introduction

Dietary diversity (DD) defined as the number of different food groups consumed over a given reference period, capable of ensuring adequate intake of essential nutrients that can promote the health, physical and mental development. Hence, lactating Women(LW) are vulnerable to malnutrition due to the physiological vulnerability that comes with childbearing is the first reason, and maternal nutrient needs increase during lactation [1].

Low Dietary Diversity results in severe problems among LW in LICs, whose diets are predominantly starchy staples. Similarly, the consumption of animal products, seasonal fruits, and vegetables are generally absent or minimal. The DD in LW is critical because they require additional energy and nutritious foods [2, 3]. LW's having a good DD, their children, adolescents, and adults may have good health, growth, and development. In a few studies, what LW consumed is strongly associated with what their children, adolescents, and adults eat [1, 4].

In Northern Ghana, 52% of LW dietary diversity scores were less than five different food groups. The five most widely consumed food groups were cereals and grains, fish and seafood, dark green leafy vegetables, species, condiments and beverages, and other vegetables apart from vitamin A rich vegetables such as tomatoes, onion, and egg-plant [5]. Besides, In West Gojam, Ethiopia, adequate DD for LW was 47.2% [6].

In Southwestern Bangladesh: a lower educational achievement, the husband is a daily wage earner, and higher household size (3 or high family members) may likely be associated with low mean DDS of LW [7]. In Northern Ghana: age, marital status, household membership structure, ethnicity, and literacy as significant socio-economic determinants of DD of LW [5]. Besides, in Aksum town, Tigray, Northern Ethiopia, reported that factors like the difference in education, feeding culture, average monthly income, not practicing home gardening, and source of drinking water were strongly associated with low DD of LW [8].

To prevent and correct a nutritional vulnerability in LW, one of the best interventions is DD and might be a means of achieving the sustainable development goals (SDGs) in an integrated manner [9, 10]. Despite a few studies on diversified nutritional requirements for LW, there are not enough studies on factors associated with low DD among LW in Ethiopia and the proposed study site. As a result, substantial numbers of LW in Ethiopia are exceedingly vulnerable to inadequate DD and nutritional deficiencies [9, 10, 11]. Therefore, this study aimed at assessing the DD and associated factors among LW in the Pawie district, Northwest Ethiopia.

## 2 Methods

### 2.1 Study area

The study was conducted in the Pawie district. It is tracking down in Benishangul-Gumuz Regional State, Northwest Ethiopia. The study area has an estimated 5, 244 square kilometers, which is located at 623 and 447 KMs away from the capital city of Ethiopia (Addis Ababa), and the regional capital city Assossa respectively. According to the 2016 Metekel zone finance and economic development projection, a total of 51,895 population are living in the district (of which 25,639 female and 26, 257 male). Administratively Pawe district has 20 (one urban and 19 rural) kebeles (the smallest administrative unit in Ethiopia). According to the 2016 bi-annual report of the Pawie district health office, there were 2,425 LW in the district. Currently, Pawe district has one general hospital, four public health centers, and 20 health posts [11].

Grains, potato, sorghum, pea and beans, and vegetables (cabbage, carrots, and tomatoes) are the primary agricultural products.

### 2.2 Study design and period

A community-based cross-sectional study was conducted from March to May 2019 to assess dietary diversity and associated factors among lactating women in Pawie district Benishangul Gumuz Regional State, Northwest Ethiopia.

### 2.3 Study population

All lactating women who lived in the Pawie district for at least six months were eligible for the study, LW who was permanently residing in the selected kebeles were considered the study units.

### 2.4 Sample size determination

In this study, the sample size was determined, by using the single population proportion formula. Taking the prevalence of good DDS as 47.8% for lactating women [1] to obtain the maximum sample size with 5% marginal error, 95% CI, a 5% non-response rate. The minimum required sample size was 403. Since it is a multistage we use 2 design effects. Then, the final sample size of this study was 806.

### 2.5 Sampling procedures

Multistage sampling technique was employed to select kebeles, households, and lactating women. From a total of 20 rural kebeles of Pawie district, five kebeles were selected by simple random sampling (lottery method) technique. LW was allocated to each selected kebeles by proportionate allocation. From each selected kebele, LW were designated by a simple random sampling method.

### 2.6 Data collection procedures

The data was collected with a face-to-face interview by the data collectors using a structured and pre-tested questionnaire. The questionnaires was adapted from English published articles and Food and Agriculture Organization (FAO) guidelines for measuring individual DD, 2011 and Household Food Insecurity Access Scale (HFIAS) to measure household food insecurity [12]. Written and structured questionnaire that consist of socio-demographic data, DD of LW and risk factors for DD used. The questionnaire had four main contents: socio-demographic data characteristics; a source of food, DD, and other individual-related factors; food security-related questions. The level of DD measured using minimum DD for a woman (MDD-W), the dichotomous indicator/tools developed by the FAO [13, 14].

A total of 16 food groups (cereals, white tubers and roots, vitamin A-rich vegetables and tubers, dark green leafy vegetables, other vegetables, vitamin A-rich fruits, other fruits, organ meat, flesh meat, eggs, fish and seafood, legumes, seeds and nuts, milk and milk products, oils and fats, sweets, spices, condiments, and beverages) considered. These food groups further regrouped into ten food groups (i.e., all starchy staples, pulses (beans, peas, and lentils), nuts and seeds, all dairy, flesh foods (including organ meat), eggs, vitamin A-rich dark green leafy vegetables, other vitamin A-rich vegetables and fruits, other vegetables, and other fruits) during analysis [3, 4].

The Minimum Dietary Diversity Score (DDS) was calculated for each LW during the previous 24 h to classify the mother's dietary diversity as good DD (≥5 food groups) or poor DD (<5 food groups) from ten food groups. Then, the outcome variable coded as a good DD ≥ 5 food groups as “1” and poor DD < 5 food groups as “0” for logistic regression analysis [12, 15].

The score of the respondents has taken, and respondents were classified as having good DD and poor DD by taking their responses if they consume greater or equal to five food groups and less than five food groups respectively [15, 16]. Then, LW who consume more than five food groups in the last 24 h classified as having good DD and poor DD otherwise. The rest questioner was adapted and modified from WHO and similar studies [4, 17, 18].

### 2.7 Data quality control

For administering the structured questionnaire, 2 Public Health Nutrition and 8 health extension workers were employed as supervisors and data collectors sequentially. The training was given for two days for both supervisors and data collectors on the objective, the relevance, confidentiality of information, respondent's right, time of data collection, and reorganization of the collected data from respective sub-cities, and submission on due time.

In addition, a pre-test was conducted on 5% of the actual sample size out of sampled kebele. The principal investigator and the supervisors checked the collected data for completeness, and corrective measures were taken accordingly. The collected data were cleaned, coded, and explored before analysis.

### 2.8 Data processing and analysis

The data were entered, coded, and cleaned using the Epi data and exported to SPSS software version 21 for analysis. Frequencies and cross-tabulations used to summarize descriptive statistics of the data. Bivariate logistic regression was employed to see the association of each variable with dependent variables. Finally, independent variables with p-values < 0.2 in the bivariate logistic regression were entered into multivariate logistic regressions to control the effect of confounding. All variables with P–values less than 5% considered to have a significant relationship with the outcome variable.

### 2.9 Ethical considerations

Ethical clearance was obtained from the institutional review board of the school of nursing, College of Medicine and Health Sciences, Bahir Dar University. The letter was submitted to Pawie district health office. An official permission letter from the office was obtained for the next steps.

The written permission letter from the district health office was submitted to each kebeles administrative office, which is the actual data collection conducted. All lactating mothers were informed about the objective of the study. Then after the objectives of the study was explained, all mothers' age was >18, and informed verbal consent from every LW was obtained. The right to participate or withdraw from the study at any time without any requirement was disclosed to the participant clearly. Furthermore, the confidentiality of the information obtained from participants was guaranteed by all data collectors and investigators by using code numbers and keeping the questionnaires locked.

## 3 Results

### 3.1 Socio-demographic characteristics of the LW

A total of 806 LW aged 15–49 years interviewed, with a response rate of 100%. Of the total LW, 50% were in the age group 20–29, 586 (72.7 %) were Amhara. Of the entire study participants, 474 (58.8%) were Orthodox. Nearly two-thirds, 493 (61.2%) LW were unable to read and write, and 570 (70.7%) participants were housewives. 521 (64.6%) of the study participants had a family member of 4–6 members, whereas 639 (79.3%) birth intervals were greater than 24 months (Table 1).

**Table 1:** Socio-demographic and economic related characteristics among lactating women (n = 806) at Pawie district Benishangul Gumuz regional state, Northwest, Ethiopia, 2019.

Background characteristics	Frequencies	Percentage (%)
**Age of the mother (in years) (n = 806)**		
<20 years	98	12.2
20–29 years	388	48.1
30–39 years	289	35.9
40 and above	31	3.8
**Ethnicity (n = 806)**		
Amhara	586	72.7
Agew	66	8.2
Kembata	60	7.4
Oromo	59	7.3
Others	35	4.3
**Religion (n = 806)**		
Orthodox	474	58.8
Muslim	256	31.8
Protestant	60	7.4
Catholic	16	2.0
**Marital status (n = 806)**		
Married	748	92.8
Divorced	50	6.2
Widowed	8	1.0
**Maternal educational level (n = 806)**		
Unable to read and write	493	61.2
Able to read and write	142	17.6
Primary school	96	11.9
Secondary and above	75	9.3
**Educational level of the father (n = 806)**		
Unable to read and write	392	48.6
Able to read and write	211	26.2
Primary	94	11.7
Secondary and above	109	13.5
**Maternal occupation status (n = 806)**		
House wife	570	70.7
Governmental employee	73	9.1
Merchant	99	12.3
Daily laborers	64	7.9
**Occupation status of the father (n = 806)**		
Farmer	522	64.8
Merchant	163	20.2
Gove employee	69	8.5
Daily laborer	52	6.5
**Income (in etb) (n = 806)**		
<500 ETB	63	7.8
500–1000 ETB	253	31.4
1001–2000 ETB	316	39.2
>2000 ETB	174	21.6
**Family size (n = 806)**		
<3 members	125	15.5
4–6 members	521	64.6
>6members	160	19.9
**Birth interval (n = 806)**		
<2 Years	167	20.7
≥2 Years	639	79.3

### 3.2 Health service utilization and child feeding of the LW

A 532 LW were getting the water from the piped source. Almost all participants had a functional toilet. 757 (95.9%) of LW had at least one Antenatal care (ANC)” follow-up, and from these greater than two-third of LW had more than three times follow-ups in the last pregnancy, but only 128 (15.9%) of LW have had Postnatal care (PNC) follow-up to the previous delivery.

Almost all LW didn't suffer from any chronic disease illness during their breastfeeding period, but only 29 (3.6%) of LW had a history of illness in the last two weeks. 676 (83.8%) of LW gave birth at a health facility, and all LW practice exclusive breastfeeding (Table 2).

**Table 2:** Health service utilization and child feeding among LW (n = 806) at Pawie district Benishangul Gumuz regional state, Northwest, Ethiopia, 2019.

Food group consumption in previous 24 h	Number	Percent (%)
**Source of drinking water**		
Piped water	532	66.0
Hand dug well	86	10.7
River	36	4.5
Protected well	148	18.4
Un protected well	4	.5
**Functional toilet service**		
Yes	792	98.3
No	14	1.7
**ANC follow up in the last pregnancy**		
Non	49	6.1
1 visit	17	2.1
2–3 Visit	201	24.9
4 and above	539	66.9
**Place of delivery**		
Health center	676	83.8
Home	130	16.2
**PNC service**		
No	678	84.1
Yes	128	15.9
**Do you have any chronic disease**		
No	802	99.6
Yes	4	.4
**History of illness in the previous 2 weeks**		
No	777	96.4
Yes	29	3.6
**Practice exclusive breastfeed**		
No	16	1
Yes	798	99

### 3.3 Dietary Diversity (DD) of the LW

A 71.6% of the LW consumed cereals, white roots, and tubers in the previous 24 h, whereas 54.1% consumed vitamin A-rich vegetables and tubers in the previous 24 h. Similarly, 50.2% consumed other fruits and vegetables in the previous 24 h. 68.1% consumed fats and oils in the previous 24 h, and two-third were organ meat and fish meat in the previous 24 h during their lactation period. Based on the categories, 297 (36.8%) of the LW had a good DDS (consumed ≥ five food groups), and 63.2% were with low DDS (consumed < five food group) (Table 3).

**Table 3:** DD among LW (n = 806) at Pawie district, Benishangul Gumuz regional state, Northwest, Ethiopia, 2019.

Food group consumption in previous 24 h	Number	Percent
**Grains, white roots and tubers, and plantains in the previous 24 h**		
No	229	28.4
Yes	577	71.6
**Pulses (beans, peas, and lentils) in last 24 h**		
No	257	31.9
Yes	549	68.1
**Other vitamin A-rich fruits and vegetables in the previous 24 h**		
No	370	45.9
Yes	436	54.1
**Dark green leafy vegetables in the previous 24 h**		
No	278	34.5
Yes	528	65.5
**Other vegetables in the previous 24 h**		
No	405	50.2
Yes	401	49.8
**Other fruits in the previous 24 h**		
No	549	68.1
Yes	257	31.9
**Meat, poultry, and fish in the previous 24 h**		
No	600	74.4
Yes	206	25.6
**Eggs in the previous 24 h**		
No	477	59.2
Yes	329	40.8
**Legumes, nuts, and seeds in the previous 24 h**		
No	513	63.6
Yes	293	36.4
**Milk and milk products in the previous 24 h**		
No	412	51.1
Yes	394	48.9
**Women DD**		
Good DD	297	36.8
Low DD	509	63.2

The cut-offs for good DD is greater or equal to five food group is good DD.

### 3.4 Food security characteristics of the LW

As shown in Table 4, Food security characteristics of the LW, from the nine HFIAS items; Only 221 (27.4%) of the LW worried about running out of food 157 (25.8%), whereas from the total 1/4^th^ of the LW were unable to eat preferred foods. 168(20.8%) of the LW was eating a limited variety of food, and only15% of LW consumed the food that they did not go to eat really, and over 1/4^th^ of LW were skipping their meals in the last 24 h. The overall prevalence of food insecurity, 334 (41.4%) of the LW was food insecure (Table 4).

**Table 4:** HFIAS items among LW (n = 806) at Pawie District Benishangul Gumuz regional state, Northwest, Ethiopia, 2019.

Characteristics	Number	Percent
**Worried about running out of food**		
No	585	72.6
Yes	221	27.4
**Unable to eat preferred foods**		
No	586	72.7
Yes	220	27.3
**Eat a limited variety of foods**		
No	638	79.2
Yes	168	20.8
**Eat foods that you did not want to eat**		
No	685	85.0
Yes	121	15.0
**Eat a smaller meal**		
No	676	83.9
Yes	130	16.1
**Skipping meals**		
No	581	72.1
Yes	225	27.9
**No food to eat of any kind in the household**		
No	754	93.5
Yes	52	6.5
**Go to sleep at night hungry**		
No	745	92.4
Yes	61	7.6
**Go a whole day and night without eating anything**		
No	761	94.4
Yes	45	5.6
**Food Security Status**		
Food secure	472	58.6
Food insecure	334	41.4

### 3.5 Factors associated with DD of the LW

Table 5 shows bivariate and multivariate analysis of factors associated with DD of LW. In the multivariate analysis, the father's occupation being a daily laborer, was two times more likely to have low DD than the counterparts [(AOR 1.820, 95% CI (. 339, 9.784)]. LW with a birth interval of less than 24 months, family size greater than six, and food insecure were 3.7, 1.5, and 2.2 times more likely, to have low DD than those with a birth interval greater than 24 months, family size less than six members and who were food secure respectively [(AOR = 3.7, 95 % CI (1.743, 7.885)], [AOR = 1.55, 95 % CI (1.046, 2.313)], and [AOR = 2.23, 95 % CI (1.626, 3.066)] respectively (Table 5).

**Table 5:** Factors associated with DD among LW (n = 806) at Pawie district Benishangul Gumuz regional state, Northwest, Ethiopia, 2019.

Variables	Poor	Good	COR at 95%CI	AOR at 95% CI
**Age of the mother (in years) (n = 806)**				
<20 years	44	54	1.94 (.852, 4.434)	1.74 (.691, 4.407)
20–29 years	238	150	.99 (.471, 2.115)	.88 (.382, 2.053)
30–39 years	173	116	1.06 (.496, 2.270)	.90 (.392, 2.054)
40 and above	19	12	1	1
**Maternal educational level**				
Unable to read and write	271	222	1.30 (.790, 2.137)	1.22 (.475, 3.132)
Able to read and write	91	51	.89 (.499, 1.584)	.76 (.297, 1.957)
Primary school	66	30	.72 (.382, 1.359)	.59 (.261, 1.320)
Secondary and above	46	29	1	1
**The educational level of the father**				
Unable to read and write	226	166	1.13 (.731, 1.739)	.52 (.223, 1.200)
Able to read and write	118	93	1.21 (.756, 1.937)	.63 (.273, 1.458)
Primary	64	30	.72 (.403, 1.284)	.51 (.232, 1.120)
Secondary and above	66	43	1	1
**Maternal occupation status**				
Housewife	317	253	1	1
Governmental employee	51	22	**1.89 (1.079, 3.313)**∗	.51 (.147, 1.793)
Merchant	61	38	1.02 (.491, 2.127)	.76 (.176, 3.260)
Daily laborers	45	19	1.47 (.753, 2.889)	.84 (.194, 3.658)
**Occupation status of the father**				
Farmer	291	231	1.00	1.00
Merchant	103	60	1.74 (.799, 3.808)	1.90 (.468, 7.725)
Gove employee	42	27	1.58 (.793, 3.154)	1.97 (.479, .142)
Daily laborer	38	14	**2.15 (1.140, 4.073)**	**1.82 (.339, 9.784)∗∗**
**The birth interval of the mothers**				
<2 Years	122	45	*2.21 (1.518, 3.218)*	*3.71 (1.743, 7.885)∗∗∗*
≥2 Years	352	287	***1***	***1***
**Income (in ETB)**				
<500 ETB	38	25	.89 (.494, 1.600)	.79 (.420, 1.496)
500- 1000 ETB	139	114	1.11 (.751, 1.636)	1.07 (.702, 1.625)
1001- 2000 ETB	197	119	.82 (.560, 1.190)	.80 (.536, 1.201)
>2000 ETB	100	74	1	1
**Marital status**				
Married	438	310	1	1
Divorced	32	18	.71 (.176, 2.852)	.60 (.141, 2.552)
Widowed	4	4	.56 (.125, 2.524)	.53 (.112, 2.555)
**Family size**				
<3 members	82	43	1	1
4-6 members	291	230	*1.09 (.729, 1.626)*	*1.23 (.693, 2.176)*
>6members	101	59	*1.02 (.718, 1.464)*	*1.56 (1.046, 2.313)∗∗*
**Food security**				
Food secure	242	230	*1*	*1*
Food insecure	232	102	*2.16 (1.61, 2.902)*	*2.23 (1.626, 3.066)∗∗∗*

∗AOR = “Adjusted odds ratio”, COR = “Crude odds

∗∗ = P < 0.05, ∗∗∗ = P < 0.01.

Bold and Italic value indicates the factors were significant from the total variables.

## 4 Discussion

Good DD for the mother's during lactation is vital for good health as well as for that their children. Various factors influence the DD of LW, and this cause for health problem of the mothers and poor growth and development of the child. According to the Essential Nutrition Action (ENA) and existing research, good DD during lactation for all LW is critical for maternal health, the health of their children, family, and nation [12]. The main aim of this study is to determine the DD and associated factors of LW in the Pawie district in Benishangul Gumuz regional state in Northwest Ethiopia.

In this finding, 36.8 % of the LW had good DD, and 63.2 % did not receive minimum DD. This is higher than the study done in Pakistan [19], Kenya [20], Ethiopia (Shashemane, Debub Bench district, and Finote Selam District, Northwest Ethiopia) [21, 22, 23]. The difference might be due to the measurement of DD, the category of food group, and the study setting. i.e., some studies used 14 food groups, and others used ten food groups consuming four and consuming five or more food groups classified as adequate DD.

This finding is almost consistent with the study done in Bale zone [10], Northeast Ethiopia [24], Aksum Tigray [25]East Gojam Zone [6], A systematic review and meta-analysis finding of Ethiopia [26, 27], and Southern Ethiopia [1]. All studies have similar sociodemographic, socioeconomic, and seasonal variations characteristics might be the possible reason for similarities.

On the other hand, the finding of this study is lower as compared with another study which is done in Nepal [28], Debre Tabor Ethiopia [29], Addis Ababa [30], and Raya Azebo Zone [31]. These might explained by the difference in the study period, which can result in food security status change, socio-demographic, socio-cultural, geographical variation (for instance, in most parts of Ethiopia produce many types of food verity like root and tuber crops, fruits, and vegetables, Teff, Degussa, cereals), while these countries may not cultivate such types of food verity, these factors are possibly caused variation in DD of LW.

In the current study, Father's occupational status being a daily laboring father, two times more likely to have low LD. Similarly, finding in Debre Tabor, Ethiopia [29], Jakarta [32], father's occupation at the lower occupational levels showed a lack of understanding of the importance of DD and reported a low DD. The true and included the late introduction of the variety of food as a food source a daily pattern. This is evidenced by the study in Ambo district West Shewa Oromia daily laborer occupation was strongly associated with mothers DD [33]. Because mothers whose husbands did daily labor did not consume the recommended dietary type since they are of low economic status, and even the mothers themselves spent most of their time in the work area as compared to the housewife.

Mothers with families seize >6 family members were 1.5 times higher risk for poor dietary diversity as compared to mothers who have less than six family sizes in this study. This is supported by the studies conducted in Wolega, Guto Gido woreda, and Nekemte referral hospital family size (AOR = 4.604, 95%CI = 1.903–11.140 have significant relation with mothers’ DD in the study area [34, 35]. When a woman had large family size, she becomes less concerned about her good DD since the food supply to the family is low and mothers worry only about what feed for her child or family than she which important determinants of her health as well as her child.

In this study, Mothers whose birth interval <24 months 3 times more likely to have a risk for poor DD as compared to their counterpart. Because, the mothers have no time to care for themselves rather they caring and worry for their little child and the next child, that may challenge their economy as well as they may have less the chances of meeting nutrient requirements for the family by improving nutritional status.

Mothers who had food insecurity were two times more likely to have poor DD as compared to the mothers who were food secured in the current study. Similarly, in a study in Mali and Debub Bench zone, women from extremely food insecure households were less likely to have good DD [22, 36]. When the woman had food security, they became more concerned with good DD, and immediately put it into practice. This is supported by a study conducted in Boston, food insecurity may worsen diet quality and health of women's in their life [37].

The husband's educational status, age, marital status, monthly income, owns agricultural land, and employment status were not associated with the DD of mothers during their lactation periods in the current study. It was disproved by a cross-sectional survey conducted in southwestern Bangladesh [7], in northern Ghana [5], In Aksum town, Tigray, Northern Ethiopia [8]. This inconsistency may be occurred due to the sample size, study setting, and statistical analysis differences.

## 5 Conclusions

Nearly two-thirds of the LW doesn't achieve the minimum DDS, and father's occupation, low birth intervals, family size being large family, and being a food-insecurity household were the significant predictors of minimum DDS in this study. Federal Ministry of Health, regional health bureaus, and agricultural government: prioritizing, planning, designing, and initiating DD intervention programs to improve maternal nutrition through appropriate food-based approaches strengthen nutrition education programs on proper maternal MDD practices and DD intake during lactation periods focused on the main predictors. In general, it is not the only task of the government; it requires multi-sectorial involvements, so every sector works cooperatively to improve the nutrition outcomes of LW. The health personals work on family planning, ANC and PNC should counsel the Women to increase the awareness of LW on how to improve the DD.

## 6 Strength and limitation of the study

The strength of this study was we used a large sample, a community-based, and required statistical analysis. However, the limitations of the study were that the data was collected by interview of the mothers about the previous 24 h of recall dietary diversity assessment, so there might be recall biases about what they consumed within the last previous 24 h [38, 39].

## Declarations

### Author contribution statement

Sileshi Mulatu, Chalachew Yenew: Conceived and designed the experiments; Performed the experiments; Analyzed and interpreted the data; Wrote the paper.

Habtamu Dinku: Conceived and designed the experiments; Wrote the paper.

### Funding statement

This research did not receive any specific grant from funding agencies in the public, commercial, or not-for-profit sectors.

### Data availability statement

Data will be made available on request.

### Declaration of interests statement

The authors declare no conflict of interest.

### Additional information

No additional information is available for this paper.

## Appendix A Supplementary data

Questionaries Questionaries
