# Supplementary material for: Dietary diversity (DD) and associated factors among Lactating women (LW) in Pawie district, Northwest, Ethiopia, 2019: community-based cross-sectional study
Source: Heliyon. 2021 Nov 27;7(12):e08495. doi: 10.1016/j.heliyon.2021.e08495 (PMC8645438; doi:10.1016/j.heliyon.2021.e08495)
Supplement: Questionaries [file mmc1.docx]

1. **Annex’s**

**Annex-I. Information sheet and consent form**

**Introduction**

Good morning/Good afternoon.

My name is ____________________ I am a member of the team for the study conducted in this area under Bahir Dar University College of Medicine and Health Science, Department of pediatrics and child health nursing. This study focused on the assessment of the dietary diversity and associated factors of lactating mothers at pawie District. The finding of this study will provide proper nutritional information and health care services to the mothers during lactation time. You are selected to participate in this study which is designed by the researcher because you fulfill all requirements to be a sample.

The data that we obtained in this interview will use only for research purposes and your response will keep confidential. For this purpose, your name will not be written here and there is no way of linking your individual responses to the final result of the study findings. The study has no risk to you except spending a maximum of 30 minutes of your time and if you face any problem in relation to the research you can contact the responsible person based on the address below. You have the right not to respond at all or to withdraw in the meantime, but your participation is highly valuable for the success of this research objective. Therefore, we politely request your cooperation to participate in this interview.

Do you agree to participate in this study? Yes, ______ continue No______

Thank you for being volunteering to participate in the study.

Investigator Name ________________________ Signature __________

Date of interview _____________, Time started ___________ end _________

**Annex II English Questionnaires**

**Part I. Socio demographic factors**

| 1 | **Characteristics of parents** | **Response and coding** | **Skip** |
| --- | --- | --- | --- |
| 101 | How old are you now? | ……..(yrs.) |  |
| 102 | What is your ethnicity | 1. Amara 2. Agew 3. Oromo 4. Tigray 5. Other (specify)……. |  |
| 103 | What is your religion? | 1. Orthodox 2. Muslim 3. Protestant 4. Others (specify) ………… |  |
| 104 | What is your marital status? | 1. Married 2. Single 3. Divorced 4. Widowed |  |
| 105 | What is your educational level? | 1. illiterate 2. Primarily Literate (1-8) 3. Secondary Literate (9-12) 4. Higher Education |  |
| 106 | What is your husband’s educational level? | 1. illiterate 2. Primarily Literate (1-8) 3. Secondary Literate (9-12) 4. Higher Education |  |
| 107 | What is your main occupation? | 1. Housewife 2. Farmer 3. Government employee 4. Non-government employee 5. Merchant 6. Daily laborer |  |
| 108 | What is your husband’s main occupation? | 1. Farmer 2. Government employee 3. Non-Government employee 4. Merchant 5. Daily laborer |  |
| 109 | Number of your Parity | ----------------- |  |
| 110 | Number of family size | …………Only |  |
| 111 | Place of delivery | 1. Health facility 2. Home |  |
| 112 | Birth order of the child? | 1. 1 2. 2-3 3. 4-5 4. 6+ |  |
| 113 | How much is your monthly income in birr? | ………..... birr |  |
| 114 | Does any member of this household own any agricultural land? | 1. Yes 2. No |  |
| 115 | If yes, how much agricultural land do members of this household own?  Local units (specify) ________ | -------------------------- |  |

**Part II. Health service utilization and child feeding Practice**

|  | **Food group consumption in previous 24 hrs** | **Response** | **Skip** |
| --- | --- | --- | --- |
| 201 | Source of drinking water | 1. Piped water 2. Hand-dug well 3. River 4. Protected well 5. Unprotected well |  |
| 202 | Functional toilet service | 1. Yes 2. No |  |
| 203 | ANC follow up in the last pregnancy | 1. Non 2. 1 visit 3. 2-3 Visit 4. 4 and above |  |
| 204 | Place of delivery | 1. Health center 2. Home |  |
| 205 | PNC service | 1. No 2. Yes |  |
| 206 | Do you have any chronic disease | 1. No 2. Yes |  |
| 207 | History of illness in the previous 2 weeks | 1. No 2. Yes |  |
| 208 | Practice exclusive breastfeed | 1. No 2. Yes |  |

***Part III.*  Dietary diversity among lactating**

| s.no | Food group consumption in previous 24 hrs | Response | Skip |
| --- | --- | --- | --- |
| 301 | Grains, white roots and tubers, and plantains in the previous 24  hrs | 1. No 2. Yes |  |
| 302 | Pulses (beans, peas, and lentils) in last 24 hrs | 1. No 2. Yes |  |
| 303 | Other vitamin A-rich fruits and vegetables in the previous 24 h | 1. No 2. Yes |  |
| 304 | Dark green leafy vegetables in the previous 24 hrs | 1. No 2. Yes |  |
| 305 | Other vegetables in the previous 24 hrs | 1. No 2. Yes |  |
| 306 | Other fruits in the previous 24 hrs | 1. No 2. Yes |  |
| 307 | Meat, poultry, and fish in the previous 24 hrs | 1. No 2. Yes |  |
| 308 | Eggs in the previous 24 hrs | 1. No 2. Yes |  |
| 309 | Legumes, nuts, and seeds in the previous 24 hrs | 1. No 2. Yes |  |
| 310 | Milk and milk products in the previous 24 hrs | 1. No 2. Yes |  |

**Part IV Food Insecurity related**

| s.no | Food insecure related questions | Responses | Skip |
| --- | --- | --- | --- |
| 401 | Worried about running out of food | 1. Yes  2. No |  |
| 402 | Unable to eat preferred foods | 1. Yes  2. No |  |
| 403 | Eat a limited variety of foods | 1. Yes  2. No |  |
| 404 | Eat foods that you really did not want to eat | 1. Yes  2. No |  |
| 405 | Eat a smaller meal | 1. Yes  2. No |  |
| 406 | Skipping meals | 1. Yes  2. No |  |
| 407 | No food to eat of any kind in the household | 1. Yes  2. No |  |
| 408 | Go to sleep at night hungry | 1. Yes 2. No |  |
| 409 | Go a whole day and night without eating anything | 1. Yes 2. No |  |

**Part V. Source of food and feeding practice**

| **Food group and feeding practice** | **Characteristics** |  |
| --- | --- | --- |
| 1. Main source of food for household (current) | 1. Own production 2. Purchasing Food aid/relief 3. Other sources |  |
| 1. Practicing home gardening | 1. Yes 2. No |  |
| 1. The daily eating pattern in the previous 7 days | 1. Three meals &/or above 2. Two meals & eating between meals 3. Two meals only or below |  |
| 1. Frequency of fruit consumption in the previous 24 hrs | 1. Not consumed at all) 2. 1–2 times 3. Three times & above |  |
| 1. Frequency of vegetable consumption in the previous 24 hrs | 1. Not consumed at all 2. 1–2 times 3. Three times & above |  |
| 1. Protein-based only in the previous 24 hrs | 1. yes 2. No |  |
| 1. Starch and protein mixed | 1. Yes 2. No |  |
| 1. Starch-based only in the previous 24 hrs | 1. Yes 2. No |  |
| 1. Dark green leafy vegetables in the previous 24 hrs | 1. Yes 2. No |  |
| 1. Vitamin A-rich fruits and vegetables | 1. Yes 2. No |  |
| 1. Other fruits and vegetables in the previous 24 hrs | 1. Yes 2. No |  |
| 1. Fats and oils in the previous 24 hrs | 1. Yes 2. No |  |
| 1. Meat and fish in the previous 24 hrs | 1. Yes 2. No |  |
| 1. Eggs in the previous 24 hrs | 1. Yes 2. No |  |
| 1. Legumes, nuts, and seeds in the previous 24 hrs | 1. Yes 2. No |  |
| 1. Milk and milk products in the previous 24 hrs | 1. Yes 2. No |  |
